# Expression of concern: Plasmonic photothermal cancer therapy with gold nanorods/reduced graphene oxide core/shell nanocomposites

**DOI:** 10.1039/d4ra90135e

**Published:** 2024-12-06

**Authors:** Kostiantyn Turcheniuk, Tetiana Dumych, Rostyslav Bilyy, Volodymyr Turcheniuk, Julie Bouckaert, Volodymyr Vovk, Valentyna Chopyak, Vladimir Zaitsev, Pascal Mariot, Natasha Prevarskaya, Rabah Boukherroub, Sabine Szunerits

**Affiliations:** a Institut d'Electronique, de Microélectronique et de Nanotechnologie (IEMN), UMR CNRS8520, Université Lille 1 Avenue Poincaré-BP 60069 59652 Villeneuve d'Ascq France sabine.szunerits@univ-lille1.fr; b Unité de Glycobiologie Structurale et Fonctionnelle (UGSF), Université Lille 1, CNRS UMR 8576 59655 Villeneuve d'Ascq France; c Danylo Halytsky Lviv National Medical University 79010 Lviv Ukraine; d Taras Shevchenko University 60 Vladimirskaya str. Kiev Ukraine; e Chemistry Department, Pontifical Catholic University of Rio de Janeiro, Rua Marques de Sao Vicente 225-Gavea Rio de Janeiro 22451-900 Brazil; f Laboratoire de Physiologie Cellulaire INSERM U1003, Equipe Labellisée par la Ligue Nationale, Contre le Cancer et LABEX (Laboratoire d'excellence), Université Lille 1 59655 Villeneuve d'Ascq France

## Abstract

Expression of concern for ‘Plasmonic photothermal cancer therapy with gold nanorods/reduced graphene oxide core/shell nanocomposites’ by Kostiantyn Turcheniuk *et al.*, *RSC Adv.*, 2016, **6**, 1600–1610, https://doi.org/10.1039/C5RA24662H.

The Royal Society of Chemistry is publishing this expression of concern in order to alert readers that concerns have been raised regarding the reliability of the data. The Royal Society of Chemistry has asked the University of Lille to investigate this matter. An expression of concern will continue to be associated with the article until we receive conclusive evidence regarding the reliability of the reported data.

Laura Fisher

5th November 2024

Executive Editor, *RSC Advances*

